# Expression of MMP-14 and its role in bone destruction in middle ear cholesteatoma: A prospective observational study

**DOI:** 10.1097/MD.0000000000035538

**Published:** 2023-10-27

**Authors:** Yu Lei, Junjun An, Qingchun Ren, Minjun Wang, Mingzhu Gao

**Affiliations:** a Department of Otorhinolaryngology, The First Affiliated Hospital of Dalian Medical University, Dalian, China.

**Keywords:** hearing loss, membrane type 1-matrix metalloproteinases (MMP-14), middle ear cholesteatoma, ossicular lesions

## Abstract

Cholesteatoma is a noncancerous cystic lesion caused by an abnormal growth of keratinizing squamous epithelium which is invasive and capable of destroying structures. A prospective study on the expression of membrane type1-matrix metalloproteinases (MMP-14) and its related influencing factors in middle ear cholesteatoma was conducted to fully understand the pathogenesis of cholesteatoma in the molecular level. We examined the expression of MMP-14 by immunohistochemical staining 39 middle ear cholesteatoma specimens and 10 external auditory meatus epithelial cell specimens. The cholesteatoma specimens were divided into 4 groups according to the degree of destruction of the ossicles during surgery. The associated factors affecting MMP-14 expression were analyzed using statistical methods; The positive expression of MMP-14 in the epithelium of the external auditory canal was significantly different between middle ear cholesteatoma and normal patients (*P* < .05); Gender, age, and the degree of hearing loss had no statistically significant effect on MMP-14 expression (*P* > .05); The expression of MMP-14 was positively correlated with the severity of bone destruction (*R* = 0.535, *P* < .05); MMP-14 plays an important role in the pathological development of the epithelium of cholesteatoma; MMP-14 expression in middle ear cholesteatoma tissue was not strongly correlated with the level of hearing loss, age or gender, but was positively correlated with the degree of middle ear bone destruction.

## 1. Introduction

A cholesteatoma is a cystic tumor of skin with keratin retention within the temporal bone. The annual incidence rate in adults is about 9/100,000.^[1]^ Current research suggests that the matrix’s enzymatic activity leads to cholesteatoma. The structures in the middle ear cleft could be destroyed by this aberrant development, which is locally invasive. Additionally, squamous epithelium may become destructive in a setting of ongoing infection, enhancing the osteolytic effects of cholesteatoma. The pathogenesis of cholesteatoma has not been fully understood. Scholars have been studying different molecular changes in recent years, including variation,^[2]^ apoptosis, inflammation, infection,^[3]^ osteolysis, lipid metabolism, and neovascularization. People discovered that the significance of numerous proteolytic enzymes should not be overlooked while investigating the damage mechanism of cholesteatoma to surrounding tissues, with MMPs (matrix metalloproteinases) becoming a research hotspot.^[4]^

MMPs can be produced by zinc calcium dependent endopeptidase in fibroblasts, keratinocytes, macrophages, or endothelial cells that have been triggered by hydrolyzed protein.^[5]^ Its main role is to destroy matrix and non-stromal cells in the extracellular matrix (ECM) in order to sustain the extracellular environment’s continuous regeneration and stability.

The membrane-bound collagenase membrane type 1-matrix metalloproteinases (MMP-14), also referred to as type 1-MMP, has 3 domains: the N-terminal, catalytic, and C-terminal. This pattern is similar for all membrane-bound MMPs which are known to target a broad range of ECM proteins. MMP-14 can drive angiogenesis in fibrin via activation of other MMPs (MMP-2, -9, and -13) as well as cytokines, chemokines, and growth factors in addition to its direct impact on matrix invasion. This worsens the breakdown of surrounding tissue proteins, compromises the structural integrity of the basement membrane, and promotes the absorption of bone. In this study, immunohistochemical labeling was used to examine the expression of MMP-14 in 39 cases of middle ear cholesteatoma and 10 cases of normal external auditory canal epithelium. Additionally, the audiological tests of patients and the clinical data of bone destruction were combined to investigate the correlation and determine the role of bone destruction in the formation of cholesteatoma epithelium.

## 2. Materials and methods

39 patients with middle ear cholesteatoma who were treated in the First Affiliated Hospital of Dalian Medical University between October 2017 and July 2018 were studied in this research. There were 17 left ears and 22 right ears, 18 males and 21 females, aged 7 to 78 years with a median age of 40 years (7–78 years). All the patients were diagnosed with acquired secondary cholesteatomas, epitympanic type. The control group was patients with tympanic membrane perforation and surgical treatment at the same stage.

The experiment was approved by the Medical Ethics Committee of the First affiliated hospital of Dalian Medical University (approval NO.2022037, Dalian, China). Written informed consent for using the samples of patients was obtained. To protect patient privacy, all patient details have been de-identified. The reporting of this study conforms to STROBE guidelines.^[6]^

### 2.1. CT scan

**Figure 1.**
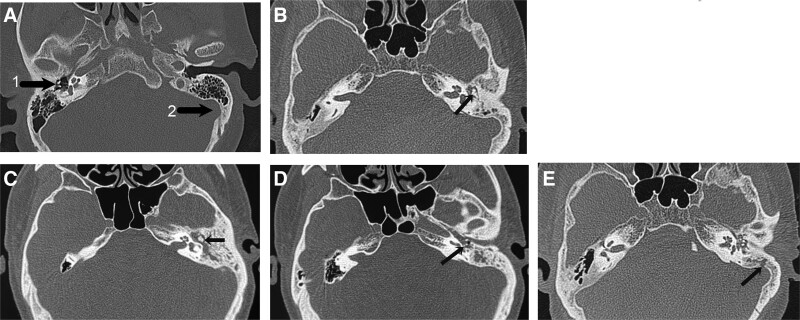
Classification of cholesteatoma according to the degree of bone destruction by CT. (A) CT image of the normal person; intact ossicle chain; intact sigmoid sinus, (B) Those without obvious destruction of auditory ossicles were graded as (+), (C) Those with destruction of incus or malleus were graded as (+ +), ↖pointed to the malleus and incus, (D) Those with destruction of incus and stapedius were graded as (+ + +), ↖ pointed to the stapedius, and (E) Those with destruction of incus, malleus, mastoid tegument and/or sigmoid sinus bone plate were graded as (+ + + +), ↖ pointed to the sigmoid sinus bone plate. (WW 3000–4000HU, WL 350–450HU).

The results of scanning and surgical records were classified according to the extent of invasion and the degree of bone destruction of cholesteatoma according to the classification of cholesteatoma on the invasion ability of middle ear cholesteatoma,^[7]^ combined with preoperative HRCT: CT image of normal persons was shown in Figure 1A; those without obvious destruction of auditory ossicles were graded as (+, Fig. 1B); those with destruction of incus or malleus were graded as (+ +, Fig. 1C); those with destruction of incus and stapedius were graded as (+ + +, Fig. 1D); patients with destruction of incus, malleus, mastoid tegument and/ or sigmoid sinus bone plate were graded as (+ + + +, Fig. 1E). The patients were divided into 2 groups according to bone destruction, they were + to + + 13 cases and + + + to + + + + 26 cases. Hearing test was performed for each patient and the air bone gap (ABG) was measured. Hearing loss was determined using the average bone conduction thresholds of 500Hz, 1000Hz, and 2000Hz, according to WHO’s Grades of Hearing Impairment^[8]^: I: 26 to 40dB for mild hearing loss (+ +), II: 41-60dB for moderate hearing loss (+ + +), I to II: 61 to 80dB for moderate and severe hearing loss (+ + + +), IV: above 80dB for extremely severe hearing loss (+ + + +), The patients were separated into 2 groups based on this standard: mild hearing loss (14 cases with I or II degree hearing loss) and severe hearing loss (14 cases with I or II degree hearing loss) (25 cases with I–II or IV degree hearing loss). In the control group, 10 cases were randomly selected and normal external auditory canal epithelium was collected.

### 2.2. Surgical approach

Canal wall up mastoidectomy with tympanoplasty was performed to each of the patient with cholesteatoma in this study. One retro-auricular incision was made. Mastoid approach with traditional milling. The posterior wall of the EAC was maintained while the facial recess, tympanic sinus, and hypotympanum were exposed to resect the cholesteoma, and all spaces were cleaned. Cartilage graft for tympanoplasty, titanium TORP or PROP prosthesis was implanted to reconstruct the new middle ear cavity. During the surgery, the capsule of the cholesteatoma was kept for pathological examination.

Tympanoplasty was performed to each of the patient in the control group. The edge of the perforation was firstly dissected and removed using a sharp pick and cup forceps. A tympanomeatal flap was created based on the location of the perforation. Implantation of temporal muscle fascia should fully cover the perforation. During the surgery, the epithelium near the eardrum was kept for pathological examination.

### 2.3. Pathological histological sections

The immunohistochemistry SP (streptavidin-perosidase) technique was employed to detect MMP-14. The paraffin-embedded tissues were sectioned at a thickness of 4 mm (Thermo Scientific HM340E paraffin section machine, USA), attached to the slide, then baked and melted wax in the oven (Thermo Scientific, USA), and incubated overnight at 60°C. Subsequently xylene dewaxing, alcohol gradient dehydration and tap water washing are performed. The processed slices were completely immersed in a pH6.0 citric acid buffer solution container, heated in a pressure cooker for 2 minutes, then removed from the container and cooled to room temperature. The tissue was washed 3 times in PBS before being inoculated with a 1:100 Rabbit anti human MMP-14 monoclonal antibody (Abcam, ab51074). MaxVision reagent (Fuzhou Maixin Biotechnology Development Co., Ltd, China, KIT-5020) was dripped and incubated at room temperature for 15 minutes after 60 minutes at room temperature. The newly prepared DAB reagent (currently used and prepared) was dripped on the tissue after washing with PBS solution, and the color development time was monitored under the microscope (about 5 minutes). Finally, all samples were dehydrated (from 70°–96°) and clarified with carboxylic acid and xylol. Then a slide adhesive for coverslip attachment was used.

### 2.4. The positive and negative control

Positive controls of MMP-14 positive breast cancer tissue sections that always have a positive reaction were used for each preparation series. PBS liquid was utilized as a negative control for the first antibody.

### 2.5. Result judgment

The protein reactants of MMP-14 were yellow and brown granules, and the staining was mainly located in the cytoplasm and membrane. Five high magnification fields were randomly selected, with no < 100 cells in each field. The positive cell rate was calculated as the percentage of cells in each field that had yellow or brown yellow granules. The average of 5 visual fields was calculated as each section’s positive rate. No staining or a positive cell rate of < 10% is negative (−), > 10% to 25% is weak positive (+), > 25% to 50% is positive (+ +), > 50% is strong positive (+ + +).

### 2.6. Statistical analysis

For statistical analysis, SPSS19.0 statistical software (IBM) was used, and χ^2^ test or Fisher exact probability test and 1 way ANOVA was used for group comparisons; the Kruskai–Wallis *H* test and Mann–Whitney *U* test were used for rank data of 2 independent samples, and the Spearman rank correlation analysis was used for correlation test. *P* < .05 indicates that the difference is statistically significant at the test level of .05.

## 3. Results

**Figure 2.**
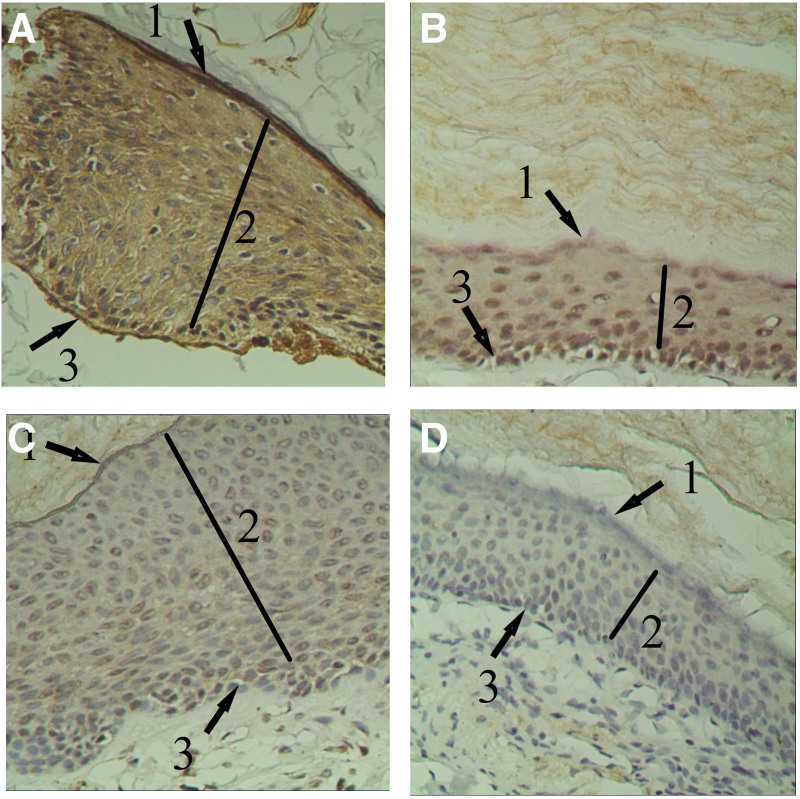
MMP-14 expression in epithelium of cholesteatoma. (A) Strong positive expression of MMP-14 (+ + +) in epithelium of cholesteatoma (×200). (B) Positive expression of MMP-14 (+ +) in epithelium of cholesteatoma (×200). (C) Weak positive expression of MMP-14 (+) in epithelium of cholesteatoma (×200). (D) Negative expression of MMP-14 (−) in epithelium of cholesteatoma (×200). Bar = 50 µm. From the surface to the bottom, the epithelium of cholesteatoma consists of the keratinized layer (1↖), the spinous layer (2↖) and the basal layer (3↖). MMP-14 = membrane type 1-matrix metalloproteinases.

MMP-14 was discovered in all cholesteatoma layers. MMP-14 was shown to be more abundant in the basal and spinous layers. MMP-14 was also discovered in some inflammatory cells in the subepithelial matrix (Fig. 2A–D).

### 3.1. The expression of MMP-14 in cholesteatoma and normal external auditory canal

**Figure 3.**
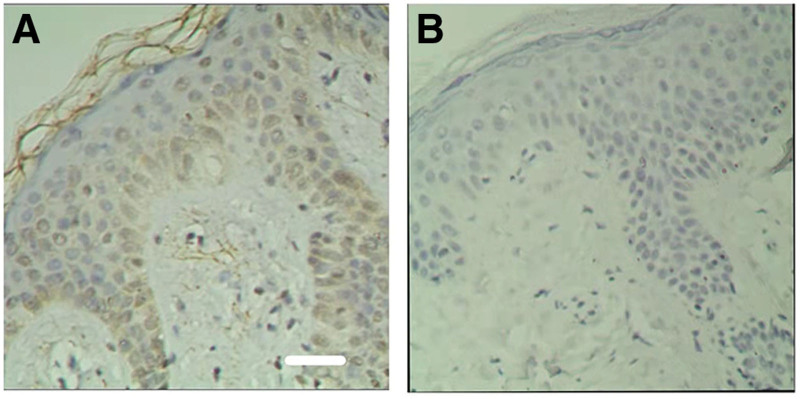
MMP-14 expression in the epithelium of external auditory canal of the control group. (A) Weak positive expression of MMP-14 (+) in epithelium of cholesteatoma (×200). (B) Negative expression of MMP-14 (−)in epithelium of cholesteatoma (×200). Bar = 50 µm. MMP-14 = membrane type 1-matrix metalloproteinases.

There were 39 cases of cholesteatoma epithelia in the experimental group, including 8 cases were strong positive (+ + +, Fig. 2A), 17 cases were positive (+ +, Fig. 2B), 10 cases were weak positive (+, Fig. 2C), and 4 cases were negative (−, Fig. 2D). In the control group, 3 cases were weakly positive (+, Fig. 3A) for the epithelium of the external auditory canal, while 7 cases were negative (−, Fig. 3B). Weak positive, positive, and strong positive were all classed as positive to facilitate statistical analysis. The distribution of MMP-14 in cholesteatoma tissue and external auditory canal epithelium was statistically significant, according to the data (Table 1).

**Table 1:** Expression of MMP-14 in cholesteatoma and normal external auditory canal.

Group	Cases of negative (%)	Cases of positive (%)	χ^2^ value	*P* value
Test group	4 (10.3)	35 (89.7)	16.318	.000*
Control group	7 (70.0)	3 (30.0)		

MMP-14 = membrane type 1-matrix metalloproteinases.

* *P* < .05, the difference was statistically significant

### 3.2. The relationship between age and MMP-14 expression

In this study, 15 of the patients with middle ear cholesteatoma were over 50 years old, while 24 were less than or equal to 50 years old. The average age was 40 years. The statistical analysis of MMP-14 detection results revealed that there was no significant difference in MMP-14 expression in patients of various ages, indicating that age had no impact on MMP-14 expression (Table 2).

**Table 2:** Expression of MMP-14 in cholesteatoma of different age groups.

Group	Total number	Cases of “−” (%)	Cases of “+” (%)	Cases of “+ +” (%)	Cases of “+ + +”(%)	χ^2^ value	*P* value
≤50 yr	24	1 (4.2)	6 (25.0)	11 (45.8)	6 (25.0)	2.939	.401
>50 yr	15	3 (20.0)	4 (26.7)	6 (40)	2 (13.3)		

MMP-14 = membrane type 1-matrix metalloproteinases.

### 3.3. The relationship between gender and MMP-14 expression

In this study, there were 18 males and 21 females with middle ear cholesteatoma. Because there was no significant difference in MMP-14 detection results between gender groups, we assumed that gender had no effect on MMP-14 expression (Table 3).

**Table 3:** Expression of MMP-14 in cholesteatoma of different gender groups.

Group	Total number	Cases of “−”(%)	Cases of “+”(%)	Cases of “+ +”(%)	Cases of “+ + +”(%)	χ^2^ value	*P* value
Male	18	2 (11.1)	4 (22.2)	8 (44.4)	4 (22.2)	0.231	.971
Female	21	2 (9.5)	6 (28.6)	9 (42.9)	4 (19.0)		

MMP-14 = membrane type 1-matrix metalloproteinases.

### 3.4. The relationship between bone destruction and MMP-14 in cholesteatoma

The Kruskai–Wallis *H* test and the Mann–Whitney *U* test were used to investigate the connection between bone destruction and MMP-14 expression. It was found that there were significant differences in the expression of MMP-14 in different degrees of bone destruction, and the expression of MMP-14 was higher in severe bone destruction group (z = −3.298, *P* = .001 < 0.05). The correlation value was *R* = 0.535, *P* = .000, showing that the amount of MMP-14 was positively connected with bone degradation, according to Spearman correlation analysis (Table 4).

**Table 4:** Expression of MMP-14 in groups with different degrees of bone destruction.

MMP-14	Degree of bone destruction		Z	r
	Cases of (+ – + +)	Cases of (+ + + – + + + +)		
−	3	1	−3.298*	0.535*
+	7	3		
+ +	2	15		
+ + +	1	7		

MMP-14 = membrane type 1-matrix metalloproteinases.

* Kruskai–Wallis H test and Mann–Whitney *U* test were used, *P* < .05, the difference was statistically significant

### 3.5. The relationship between hearing and MMP-14

There were 14 cases of grade I to II hearing loss and 25 cases of grade I to II to V hearing loss in the experimental group. The association between hearing loss and MMP-14 expression was investigated using the Kruskai–Wallis H test and the Mann–Whitney *U* test. The expression of MMP-14 was found to be higher in the I to II to IV hearing group (z = −2.34, *P* = .019 < 0.05, Table 5), that was statistically significant. However, when we did the correlation analysis for hearing level and MMP-14 expression, we found they were not correlated. The relationship between hearing loss and MMP-14 was studied using Spearman analysis, and the correlation coefficient was *R* = 0.38, *P* > .5. It is suggested that the expression of MMP-14 is not closely related to hearing impairment. One way ANOVA was used to compare the relationship between different expression levels of MMP-14 and ABG (Table 6). The results showed that there was no significant difference between the groups (*P* > .05). ABG did not change with the expression of MMP-14.

**Table 5:** Relationship between the expression of MMP-14 and the grade of hearing loss.

MMP-14	Hearing loss classification		z	r	*P* value
	Cases of level I–II (%)	Cases of level I–II–IV (%)			
−	3 (7.7)	1 (2.6)	−2.34	0.38	> .05
+	6 (15.4)	4 (10.0)			
+ +	3 (7.7)	14 (35.9)			
+ + +	2 (5.1)	6 (15.4)			

MMP-14 = membrane type 1-matrix metalloproteinases.

**Table 6:** Relationship between the expression of MMP-14 and ABG.

Mmp-14	n	Mean	Std. Deviation	F	*P* value
−	4	29.0000	6.48074	1.212	.320
+	10	31.7000	6.78315		
+ +	17	29.7647	6.36916		
+ + +	8	35.3750	9.95615		

ABG = air bone gap, MMP-14 = membrane type 1-matrix metalloproteinases.

## 4. Discussion

MMP-14 is a kind of membrane matrix metalloproteinase with a significant extracellular matrix degradation effect.^[9]^ Its substrates include collagen I, II, I to II, fibronectin, CD44, and so on. It can also activate other members of the MMP family,^[10]^ induce angiogenesis,^[5]^ and enhance cell metastasis.^[11]^ At present, it is speculated that MMP-14 leads to bone invasion of cholesteatoma through the following ways: Activation of MMP-2: MMP-2 is type IV collagenase, which can degrade laminin and fibronectin of basement membrane. Keratinocyte invasion after the basement membrane damaging is a major etiology of cholesteatoma.^[12]^ MMP-14 hydrolyzes the pro peptide of MMP-2 proenzyme to generate the precursor of MMP-2 during the cholesteatoma process. The precursor next hydrolyzes to produce active MMP-2, providing a basis for bone degradation;^[13,14]^ Activation of MMP-13: MMP-13 can activate osteoclasts and trigger a cascade reaction with MMP-2, exacerbating bone degradation;^[15]^ Promoting angiogenesis: the expansion and growth of cholesteatoma requires abundant blood supply to provide nutritional conditions for its continuous proliferation. MMP-14 promotes angiogenesis mainly by degrading extracellular matrix, producing angiogenesis-promoting silver, and degrading antivascular factors.^[16]^ Its function in stimulating angiogenesis in fibrin is significantly stronger than that of serine, cysteine and other MMPs.^[17,18]^

According to the findings of this study MMP-14 was found in all layers of the cholesteatoma epithelium with the strongest expression in the basal layer and the adjacent spinous layer (Fig. 2A). Because the basal layer is equivalent to the germinal layer of epithelial tissue, and its proliferation is very active. The adjacent spinous layer is formed by the continuous differentiation and proliferation of basal cells. This phenomenon indicated MMP-14 might involve in basement membrane degradation and stimulated cell proliferation. When MMP-14 breaks down ECM, including basement membrane, the extracellular matrix loosens, allowing basal cells to proliferate. The high production of MMP-14 can be stimulated by inflammatory reactions and different cytokines in the ECM, resulting in a vicious cycle of matrix destruction cell proliferation.^[19]^ According to the immunohistochemical localization, the positive reaction granules of MMP-14 were mainly located in the cytoplasm and cell membrane. Due to the role of hydrophobic transmembrane area in the structure of MMP-14, the transmembrane of MMP-14 was mainly located on the cell membrane after maturation, which was also the reason for the staining of MMP-14 cytoplasmic envelope. The characteristic of MMP-14 locating cell membrane also increases the contact between MMP-14 and ECM, thus accelerating the degradation process. In this study, we analyzed the level of MMP-14 and the degree of bone destruction, consists with above theory, we found that there was a positive correlation between them.

Bone destruction, especially the discontinuity of ossicular chain, greatly affects the hearing of the patients. The expression of MMP-14 is positively correlated with the bone destruction of the ossicles. However, we didn’t find any relationship between MMP-14 and hearing, we speculate that it might due to a variety of factors affecting the hearing of patients; Whether there is an acute infection or not: when cholesteatoma of the middle ear is not worsened by infection, the hearing loss is not visible or simple conduction deafness. Inflammatory mediators and bacterial exotoxins generated by inflammatory stimulation can reach the inner ear through the cochlear window, destroying hair cells and causing sensorineural hearing loss when combined with infection; Site of bone destruction: some studies have found that bone erosion away from the auditory ossicular joint in some patients, including the malleus head, the long foot of incus and even a small foot of stapes, did not cause hearing loss;^[20]^ Presbycusis, history of ototoxic drug use, virus infection, autoimmune disease, sudden deafness and so on.

In this experiment, only 3 samples of the control group showed weak positive reaction, which showed that the expression of MMP-14 was weak in the epithelium of normal external auditory canal. In fact, MMP-14 is widely distributed in the human body, which is regulated by various mechanisms. It participates in the degradation of physiological matrix and is necessary for normal physiological activities. Some animal experiments have shown that MMP-14 knockout mice will have physical shrinkage, bone deformity, death and other characteristics,^[21]^ indicating the importance of MMP-14 in physiological activities. However, the expression level is quite low under normal physiological conditions, and it will only increase when pathological processes like as inflammation, malignancy, and neovascularization.^[14]^ We did statistical analysis based on age and gender, and found that MMP-14 expression in the 2 groups was not statistically significant, indicating that MMP-14 expression is unrelated to age and gender.

The difference in MMP-14 expression in cholesteatoma tissue and normal skin of the external auditory canal was detected using an immunohistochemistry technique in this study. Blot and gelatin zymography were used to detect MMP-14 expression in tissues, as well as the difference between proenzyme and active enzyme expression, in order to better understand MMP-14 expression in middle ear cholesteatoma and lay a foundation for clinical application of MMP-14 indicators in the evaluation of disease progression or surgical prognosis. Due to the limited participants, the number of the selected subjects was small in this study. Increased activity of MMP-14 has been demonstrated in cholesteatoma tissue; however, the underlying molecular mechanism has not been investigated in detail. Thus, further surveys for the subjects in this study will be conducted to confirm the findings of the present study.

## 5. Conclusion

Our study showed the expression of MMP-14 in cholesteatoma epithelium was significantly higher than that in normal external auditory canal epithelium which indicated MMP-14 might play an important role in the pathological development of cholesteatoma. MMP-14 expression was not associated to age or gender; however, it was linked to bone degradation, according to statistical analysis. The level of MMP-14 in the sever hearing loss group was higher than that in the mild hearing loss group, but there was no correlation between the expression of MMP-14 and hearing.

## Author contributions

**Conceptualization:** Mingzhu Gao.

**Data curation:** Yu Lei, Junjun An, Qingchun Ren.

**Formal analysis:** Junjun An, Minjun Wang.

**Project administration:** Mingzhu Gao.

**Supervision:** Mingzhu Gao.

**Writing – original draft:** Yu Lei, Qingchun Ren.
